# Alcoholism and Phthisis

**Published:** 1911-05

**Authors:** 


### Alcoholism and Phthisis.

Dr. Thomas D. Lister writes in the *Medical Press and Circular* on certain conditions which bear on the prognosis of phthisis. He is of the opinion, which is fortified by an experience of many years, that among the most common conditions found associated with consumption is that of alcoholism. At one time, and not so long ago, it used to be the custom to give alcohol in somewhat large quantities to phthisical patients. The administration of alcohol combined with overfeeding used to be the routine practice in Nordrach Sanatorium; this, however, is now discontinued. In the opinion of Lister, alcoholism is very closely allied to the causation of tuberculosis, and in the prognosis of the disease he thinks it may be accepted as an axiom that the chronic alcoholic does not materially benefit by any amount of treatment. For instance, studies of mortality statistics show that the list of occupations suffering most from alcoholism almost coincides with the list of occupations in which phthisis figures most significantly. Thus in the liquor trade the incidence of pulmonary tuberculosis is most conspicuous. In short, the abuse of alcohol conduces to the diminution of the resistance of the individual to the tubercle bacillus. Of course, the life led by the bartender is inimical in most respects to health, the confinement, the oftentimes foul air, the spitting, and the dirty habits of many of the people with whom he is thrown into contact are all factors to be taken into consideration and all tend to produce a lowered vitality. But in any occupation in which the abuse of alcohol is practiced there is invariably a high mortality from tuberculosis, and the statement that there is a very distinct relationship between alcoholism and pulmonary tuberculosis, Lister claims, is fully shown by mortality statistics.—*Exchange.*

